# A case study approach to the learning effects of self-assessment in translation learning: evidence and mechanism

**DOI:** 10.3389/fpsyg.2025.1692773

**Published:** 2026-01-08

**Authors:** Tiantian Wang, Gang Zeng

**Affiliations:** School of Foreign Languages, Dalian Maritime University, Dalian, China

**Keywords:** cognitive psychology, metacognition, scaffolded learning, self-assessment, self-regulated learning, translation education

## Abstract

While self-assessment has been widely studied, its cognitive-psychological mechanisms–particularly in translation learning–remain underexplored, especially within China’s higher education context. This study addresses this gap by investigating how learners generate feedback through scaffolded comparisons during self-assessment, a process central to metacognition and self-regulated learning. An English-major undergraduate with documented advanced translation competence and feedback literacy, established through 2 years of classroom observation, was purposefully selected for this case study, which tracked her interactions with multiple scaffoldings across iterative self-assessment cycles. Thematic analysis of interviews, combined with fine-grained analysis of translation products and scaffolding use, reveals that learners engage in dynamic comparisons between their outputs and available inputs, triggering learning effects. Crucially, the interactionist perspective frames these comparisons as psychologically meaningful interactions between learner beliefs and external supports, elucidating the cognitive mechanism behind self-assessment efficacy. By bridging cognitive science (metacognitive processes) and educational psychology (learner autonomy), this research advances process-oriented assessment models while hopes to contribute to mental well-being through its implications for reducing academic anxiety. More broadly, the findings speak to the potential of psychology-informed strategies in translation education to support the integrated development of professional competence and lifelong learning skills.

## 1 Introduction

Over the past two decades, research has extensively examined how interaction fosters language learning opportunities (Bllaca and Villarreal, 2025; Adams and Oliver, 2023). From an interactionist perspective (Long, 1996), self-assessment (SA)–a metacognitive process of generating internal feedback by evaluating one’s cognitive processes and outputs against standards (Butler and Winne, 1995; Panadero et al., 2016)–represents a critical yet underexplored dimension in translation education. Although key to autonomous learning and learner-centered assessment (Iglesias Pérez et al., 2020), its cognitive-psychological mechanisms are poorly understood, particularly in China’s higher education where it is often used as a prescriptive tool without attention to its psychological underpinnings.

The current study addresses two critical research gaps:

(1)    The lack of mechanistic understanding regarding how learners generate feedback through scaffolded comparisons during SA;
(2)    The absence of longitudinal research combining think-aloud protocols with detailed analysis of learning artifacts to capture real-time metacognitive activity.

This study therefore employs a longitudinal case study approach to track an English-major undergraduate’s interactions with multiple scaffoldings across iterative SA cycles. The participant–purposefully selected from the author’s teaching context after over 2 years of observation for her developed feedback literacy–provides a representative case for an in-depth examination of the core challenges students face when processing feedback. Through a thematic analysis of rich interview data, coupled with a meticulous examination of her translation drafts and scaffolding use patterns, this research aims to illuminate the dynamic, internal cognitive comparisons that drive learning. By offering a thick description of this specific case, the study seeks to generate transferable insights, positing that a nuanced understanding of such individual learning dynamics is a crucial step toward effectively leveraging SA to enhance translation competence both within and beyond the classroom.

## 2 Literature review

### 2.1 Self-assessment studies in translation classroom

The evolution from teacher-centered to learner-centered paradigms in education has gained significant traction across disciplines, particularly in translation instruction where the cognitive complexity and ill-structured nature of translation problems demand self-regulated learning approaches (Alves and Albir, 2024; Sirén and Kai, 2002). This shift is further justified by the inherent subjectivity of translation products, which rely heavily on translators’ decision-making processes (Pym, 2003), and the growing need for adaptive skills in a dynamic job market (Pietrzak, 2018). Within this context, self-assessment (SA) emerges as a fundamental component of learner-centered assessment, offering not only cognitive benefits but also significant emotional advantages, including enhanced motivation (Su, 2023), increased confidence, and improved self-efficacy (Hung, 2019). The divergent nature of translation tasks makes SA particularly suitable, as it generates multiple learning opportunities through varied feedback practices (Yan and Carless, 2021).

Extensive research has examined SA accuracy, based on the premise that accurate self-assessment is essential for identifying strengths and weaknesses (Dochy et al., 1999) and for drawing valid conclusions about work quality (Kane, 2006; Messick, 1989). Studies typically measure SA accuracy by correlating self-ratings with external assessments, such as language tests or teacher evaluations (Oscarson, 1989), the latter being more prevalent in translation contexts (Zheng et al., 2021). In translation SA research, Campbell (2014) identified significant gaps between students’ self-ratings and external assessments, with lower-level students tending to overestimate and higher-level students underestimating their performance. These results highlight the value of scaffoldings such as rubrics. Among these, Robinson et al.’s (2006) five-level SA rubric–which includes criteria such as source text comprehension, vocabulary, grammar, sentence structure, terminology, cohesion, and fluency–has demonstrated strong validity and applicability in English-targeted translation tasks.

A second line of inquiry has examined the effects and optimization of SA in translation learning. Empirical studies indicate that SA promotes learner autonomy learner autonomy (Galán-Mañas, 2016), fosters positive learning attitudes (Li, 2018), and enhances self-awareness and reflection (Han and Fan, 2020). The development and application of translation rubrics have been found to amplify these benefits (Huertas-Barros et al., 2018; Mei and Chen, 2025), establishing SA as a valuable pedagogical tool. However, realizing such effects depends on specific prerequisites–particularly the use of effective assessment criteria to guide structured self-assessment. Although progress has been made, optimal SA procedures remain underexplored, representing a significant gap in the literature.

Recent work has adopted process-oriented approaches to examine SA in translation. Fernández and Zabalbeascoa’s (2012) metacognitive framework marked a key shift by conceptualizing translation as a set of complex cognitive sub-processes–such as reading, comprehension, and semantic transfer–that depend on high-level metacognition. By broadening SA to include not only accuracy but also metacognitive reflection, and by treating SA as dynamic rather than static, their study laid a foundation for subsequent research. Through qualitative analysis of resolved translation problems and learners’ metacognitive justifications, they introduced a novel means of assessing SA quality and process.

Nonetheless, the field still lacks fine-grained case studies tracing the SA process and clarifying its underlying learning mechanisms in translation. Although prior research has confirmed the benefits of SA and investigated its accuracy and outcomes, few studies have delved into the cognitive-psychological processes through which SA enhances translation learning. This gap is especially evident in China’s higher education context, where little is known about how learner beliefs interact with external scaffoldings during SA. Addressing this limitation, the present study investigates the dynamic comparisons and feedback generation processes central to effective SA, aiming to connect cognitive science with educational psychology to inform both theory and practice in translation pedagogy.

### 2.2 Self-assessment research in disciplines beyond translation

Although self-assessment (SA) has been widely investigated across higher education disciplines, its influence on linguistic skill development–particularly in translation learning–remains relatively under-researched and inconclusive (Huisman et al., 2019). Common SA tools such as learning journals and numerical scales are often recommended to foster reflective competence. Yet, the relationship between SA and self-reflection–which jointly shape SA quality–calls for further investigation in translation settings. This section synthesizes empirical evidence from other fields to inform the application of SA in translation studies, with a focus on two core aspects: scaffolded SA methodologies and the development of feedback literacy.

#### 2.2.1 Scaffolded self-assessment and feedback literacy

A sociocultural viewpoint (Vygotsky, 1978) considers learning as a socially bound activity, where interaction, particularly between an expert and a novice, is pivotal for cognitive and language development. Thus, scaffolded approaches have gained broad acceptance as a means to address challenges in student SA performance (Su, 2021). Rubrics, the most prevalent scaffolding tool, serve a dual function: they clarify competency expectations and provide detailed performance descriptors, supporting both learners and instructors in evaluating task completion. However, the abstract nature of rubrics can limit their effectiveness, leading to the incorporation of exemplars to make assessment standards more tangible (Hendry et al., 2012; Carless, 2020). The combined use of rubrics and exemplars expands students’ sources of feedback; comparisons between drafts and exemplars have proven especially effective in stimulating internal feedback (Nicol and Selvaretnam, 2021). Empirical evidence indicates that SA are most beneficial when students produce initial drafts and then engage in iterative comparisons with multiple scaffolding resources (Hill and West, 2020; Nicol, 2021).

Feedback, understood as information regarding performance or understanding (Hoo et al., 2022), enables learners to recognize discrepancies between their current and target competency levels (Hattie and Gan, 2011). However, feedback alone is not enough. Recent research highlights the need to develop feedback literacy–defined as the ability to interpret, process, and act upon feedback (Boud and Molloy, 2013; Carless and Boud, 2018). From a sociocultural perspective, feedback is viewed as a dialogic process that depends on meaningful teacher–student interaction to achieve shared understanding (Ajjawi and Boud, 2018). External feedback serves as a scaffolding that supports error identification and revision (Merkel, 2018) and is essential for building feedback literacy (Mak, 2019). Although immediate instructor feedback following SA is recommended, this practice has received limited attention in translation research. Given the cognitive complexity of translation and SA, case studies incorporating think-aloud protocols (Tan and Cho, 2021) offer a promising approach for elucidating these interactive processes.

#### 2.2.2 Synthesis and implications for current research

Key implications for enhancing self-assessment (SA) in translation can be summarized as follows: (1) the combined use of multiple scaffoldings–such as rubrics, exemplars, and iterative comparisons–enriches learning opportunities; (2) timely teacher feedback following SA deepens reflection and strengthens feedback literacy; and (3) methodological approaches such as case studies and think-aloud protocols help uncover the cognitive-psychological mechanisms underlying SA.

**FIGURE 1:**
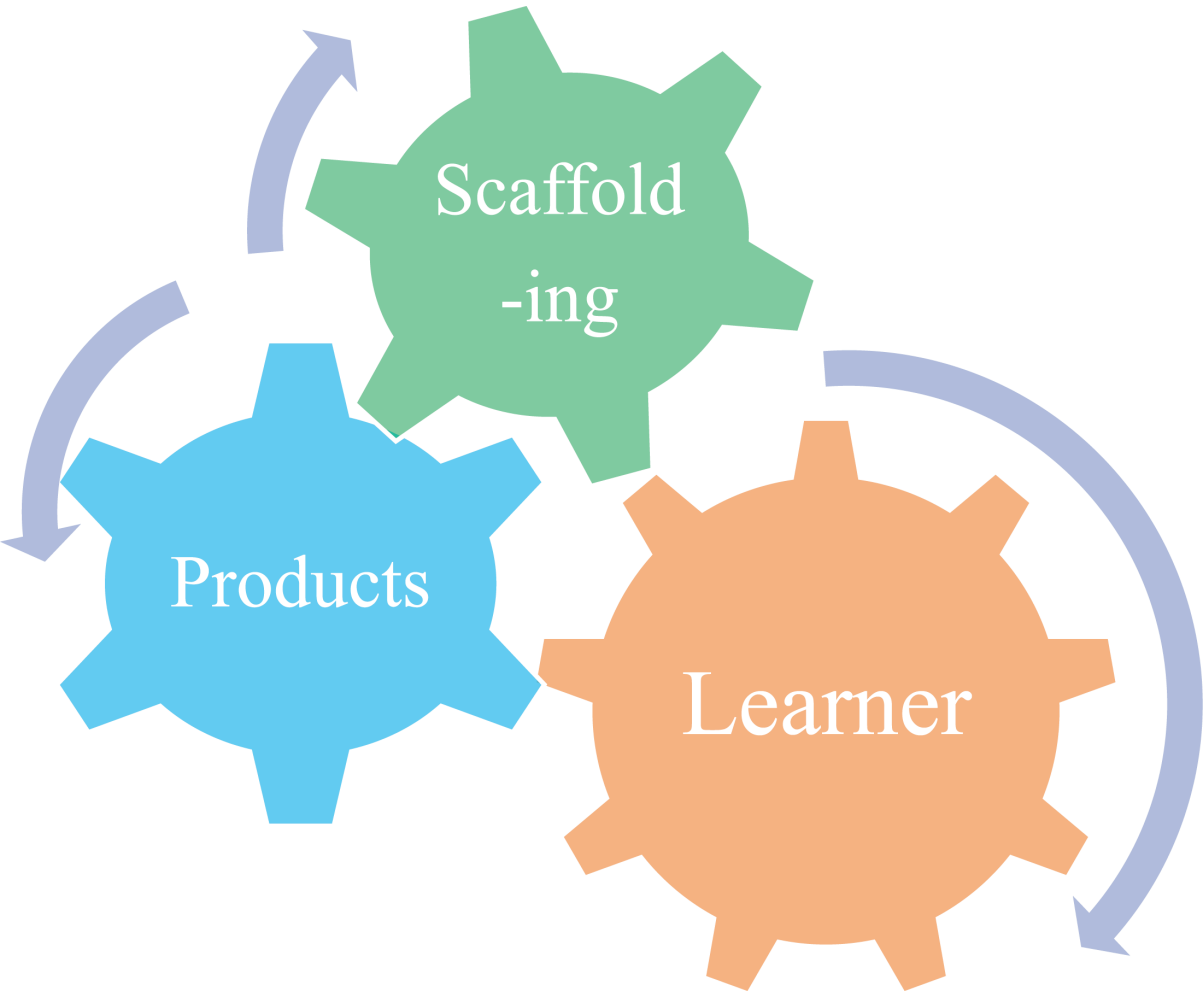
A conceptual model of self-assessment for learning as a learner-driven resonance between learning products and scaffoldings. This model illustrates how learning outcomes emerge through dynamic interactions between the learner’s activities and instructional scaffoldings, with the learner’s attributes centrally influencing the entire process. The bidirectional arrows represent the resonant interaction between scaffoldings and learning products, moderated by the learner’s performance.

These principles underpin the conceptual model presented in Figure 1, in which learners iteratively compare their drafts with scaffoldings, mediated by their pre-existing translation beliefs, to promote learning. By addressing key gaps in SA research, this study proposes a psychology-informed framework for translation, one that may contribute to broader educational objectives aimed at fostering metacognitive development and supporting mental well-being.

## 3 Methods

Specifically, this study sought to answer the following research questions:

(1)    What types of learning evidence emerge during SA in translation learning?
(2)    What cognitive-psychological mechanisms underlie the effectiveness of SA for translation learning?

To address the research objectives–which focus on tracing the cognitive and interactive processes underlying self-assessment (SA) in translation–a qualitative single-case study design was adopted. This approach is particularly suited to capturing the complexity and dynamics of how a learner engage with scaffolded SA across multiple cycles (Duff, 2007), especially when the aim is theory elaboration rather than statistical generalization (Seawright and Gerring, 2008). In this study, an in-depth examination of a single learner’s SA behaviors, beliefs, and feedback processing was deemed not only appropriate but necessary to identify nuanced mechanisms often obscured in larger-scale studies.

### 3.1 Participant

This study was conducted at a prominent foreign language university in China. The participant, a 20-years-old third-year English major and native Mandarin speaker, was purposively selected in accordance with the case selection criteria of typicality and extremeness (Seawright and Gerring, 2008). She had no prior experience with self-assessment (SA). Two translation tasks (Task 1 and Task 2) were administered to assess her translation competence, on which she scored 3 and 3.5, respectively, on a 5-point scale. She thus represents a learner profile characterized by strong translation ability–key attributes for examining SA processes under supportive conditions.

Her language proficiency is reflected in outstanding performance on standardized tests: she passed TEM-4 (Test for English Majors-Band 4) and achieved a score of 580 on CET-6 (College English Test-Band 6). Having received 12 years of formal English instruction, she has consistently excelled in translation practice and theory courses, earning grades above 90 out of 100 for two consecutive semesters (details are provided in Supplementary Appendix A). She actively participates in class discussions, exhibits well-developed metacognitive awareness, and has received award in several translation competitions. These accomplishments signify high learning motivation and advanced prior knowledge in translation.

As a highly proficient learner, she constitutes an ideal case for exploring the core mechanisms and persistent challenges of SA. Her profile helps ensure that any observed difficulties arise from the intrinsic complexity of SA’s cognitive and metacognitive demands, rather than from deficiencies in translation knowledge or experience.

### 3.2 Procedures

**FIGURE 2:**
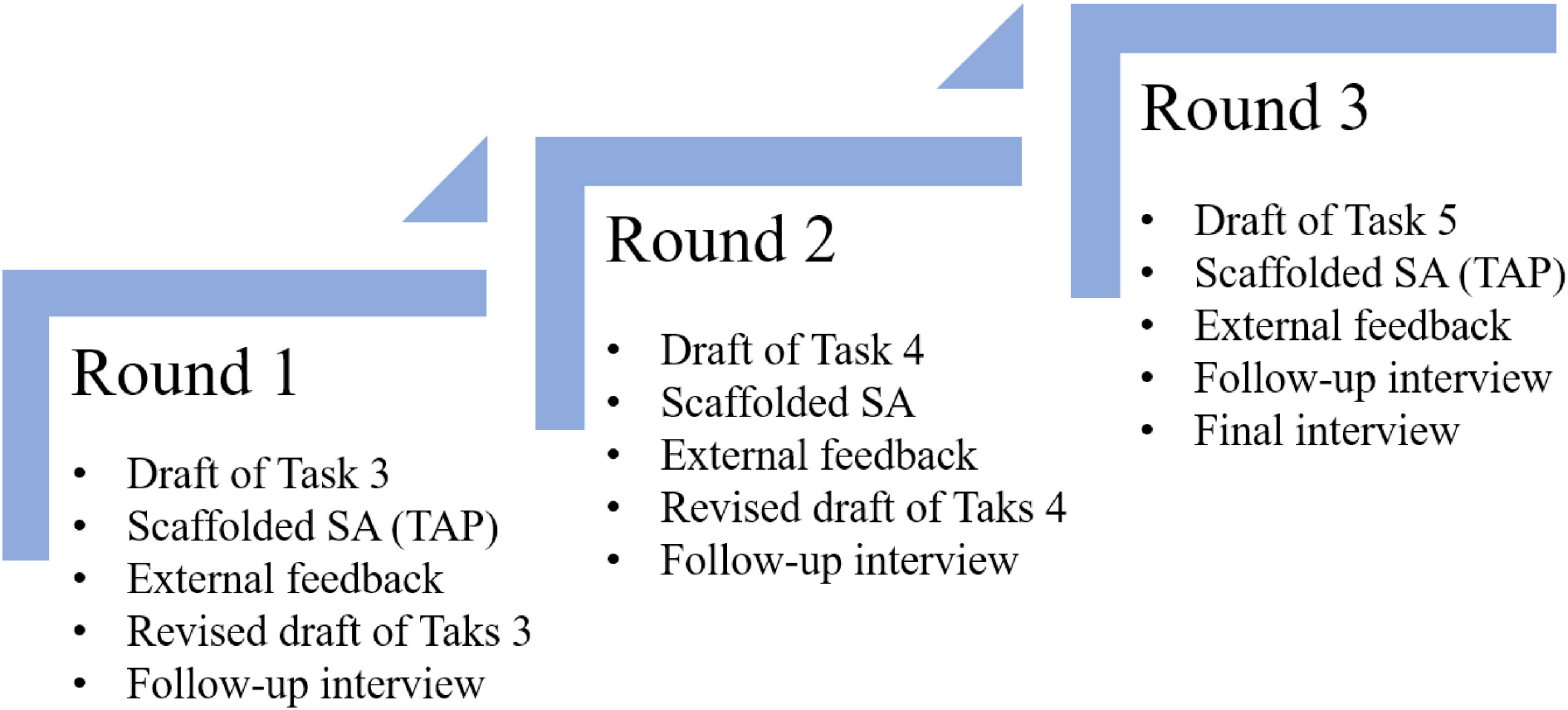
Data collection procedures. Over three consecutive weeks, the participant sequentially engaged in a cyclic process of completing a translation task (see Supplementary Appendix D for details), conducting self-assessment, receiving external feedback, revising the translation, and participating in a retrospective interview. This design allowed for a comprehensive portrayal of the learner’s holistic SA experience.

Data collection spanned 3 weeks. Initially, signed consent forms were obtained from the participant. Then, to test the feasibility of translation SA, the participant completed a piloting (see Supplementary Appendices F, H, M for details) session 1 week before the actual data collection. The data collection process is summarized in Figure 2, designed to capture the dynamic interactions between learner cognition and instructional scaffolding. In the first two phases, the participant completed translation tasks, followed by scaffolded SA using: (1) a reference translation (see Supplementary Appendix E for details), (2) a five-level SA rubric (see Supplementary Appendix B), and (3) teacher feedback–provided by the researcher to facilitate in-depth observation. Each phase concluded with draft revisions and semi-structured interviews. The third phase introduced a controlled condition, utilizing only the reference translation and rubric to isolate the effects of scaffolding.

Three follow-up interviews and a final interview were administered. Follow-up interviews aim to (1) improve data clarity or fill in missing data, and to (2) collect the participant’s authentic perceptions. The final interview was designed to gather the participant’s general perceptions. All interviews were recorded and transcribed verbatim. In summary, multiple data sources served as a mini-portfolio to keep track of the participant’s translation practice and SA performance.

Data collection across all phases integrated multiple sources:

- Think-aloud protocols, to capture real-time metacognitive processes during SA;
- Semi-structured interviews (post-SA), exploring reflective depth and belief development;
- Product analysis, including translation drafts and rubric-based self-ratings;
- Reflective journals, collected via self-designed SA questionnaires (see Supplementary Appendix C for details) that prompted identification of strengths, weaknesses, difficulties, and learning insights.

It is important to note that the researcher also served as the participant’s instructor for over 2 years. While this prolonged engagement afforded valuable contextual understanding, it also introduced the potential for observer bias due to the dual role of teacher and researcher. To mitigate this, a reflective journal was maintained throughout the research process to critically examine assumptions and the potential influence of the researcher–participant relationship on data interpretation. For example, when analyzing the participant’s difficulties with feedback, the researcher deliberately contrasted instructional perspectives with the participant’s reported experiences to ensure findings were grounded in the data rather than preconceived expectations.

This multi-modal design enabled triangulation across behavioral (revisions), cognitive (think-aloud data), and affective (interview reflections) dimensions. The phased structure allowed for the observation of developmental trajectories across successive SA cycles, while controlling for variations in scaffolding. All procedures adhered to process-oriented assessment principles, emphasizing the interplay between learner internalization (self-ratings and reflections) and external supports (rubrics and reference translations).

Overall, the methodology was designed to operationalize the theoretical focus on metacognitive comparison processes, while addressing the specific context of Chinese higher education through culturally relevant data collection tools. The longitudinal design provided a robust empirical basis for modeling the cognitive mechanisms of SA in translation pedagogy.

An overview of the case study is shown in Table 1. The subject was required to participate in three SA activities within three consecutive weeks.

**TABLE 1:** Overview of the case study.

No.	Collected data	Analytical contribution
1	Translation scripts and revised scripts	Collecting learning evidence
2	TAPs recording of translation process and SA process	Tracking translation SA procedures and patterns
3	Reflection journal of translation process and SA process	Revealing key elements in translation SA
4	External feedback script and recording	Observing interactions between learner and scaffoldings and tracking improvement
5	Follow-up interviews recordings	Clarifying confused learner behaviors and documenting immediate learner feelings (e.g., I noticed that after Task 3, you went to the reference translation first. Could you walk me through your decision to do that, instead of checking the rubric?)
6	Final interview recording	Recording learner’s changes on SA, translation belief and future learning (e.g., If you had to explain self-assessment to another student, what would you say?)

### 3.3 Data analysis

The analysis combined thematic analysis (Braun and Clarke, 2006) of verbal protocols and interviews to identify emergent student feedback patterns and Microgenetic analysis (Siegler, 2006) of translation scripts to track competency development so as to unravel the belief-scaffolding dynamics.

#### 3.3.1 Thematic analysis

Guided by an adaptation of De Kleijn’s (2021) feedback process model, the thematic analysis conceptualized SA as a dynamic cognitive system comprising four sub-processes (see Supplementary Appendix G for the codebook): (1) Meaning Construction: how learners interpreted rubric and exemplar scaffoldings; (2) Strategic Application: translation choices triggered by self-generated feedback; (3) Behavioral Response: draft revisions serving as evidence of belief adjustment; (4) Active Seeking: patterns of spontaneous scaffolding utilization.

To ensure coding reliability, the researcher and another coder coded approximately 20% of the whole data set independently (inter-coder agreement 0.85), with the consensus reached through face-to-face negotiation and involving the third coder where necessary. After the consultation, the two coders coded another 10% of the data, and the coding agreement rose to 0.94. Essential consultation results were documented when necessary. Examples of thematic analysis are shown in Table 2.

**TABLE 2:** Thematic analysis examples.

Round	Excerpts	Codes (subcodes)	Definitions
1	Ugh, I should’ve gone with “commuter.” I just didn’t know the right word. I had to fall back on a literal translation here because I was stuck. If I had a computer on hand, I’d definitely look it up.	Making sense of feedback information (comparing)	Comparing what can I learn from these comparisons?
2	I really need to work on learning more words with multiple meanings, phrases, and synonyms.	Using feedback information (action planning)	What do I have to do to use the feedback information?
3	I can’t believe I forgot again!	Responding to feedback information (addressing emotional impact)	What was the emotional impact of the feedback information?
4	How can I get better at learning expressions in context? Any tips?	Seeking feedback information (learning objective-based feedback request)	Could you give me feedback on this?

#### 3.3.2 Product-traceability analysis

Based on a product-traceability analysis that triangulates multiple data sources–including initial and revised drafts (see Supplementary Appendix I for details), teacher (see Supplementary Appendix K for details) feedback records, self-assessment (SA) reflection (see Supplementary Appendices J, L for details), think-aloud transcripts, and follow-up and final interviews–this study identifies key linkages across SA sub-phases and elucidates how SA facilitates translation learning.

Examples of product-traceability analysis is shown in Table 3.

**TABLE 3:** Product-traceability analysis examples.

No.	Content from TAPs	Source of feedback	Target use of feedback
1	Tense consistency should be noted, and here should be present perfect tense.	Week 2 teacher feedback	Translating
2	Sentences should be processed flexibly; for example, a topic sentence can be added to achieve the purpose of simplification and logical clarity.	Week 1 internal feedback	Self-assessing

## 4 Results

The study yielded three key findings: (1) the evolution of the student’s feedback process pattern; (2) the visualization of cross-round interactions and their implications for learning; and (3) the demonstration of learning effects through exemplary SA-based learning chains extracted from the data.

### 4.1 Evolution of the student’s feedback process pattern

**FIGURE 3:**
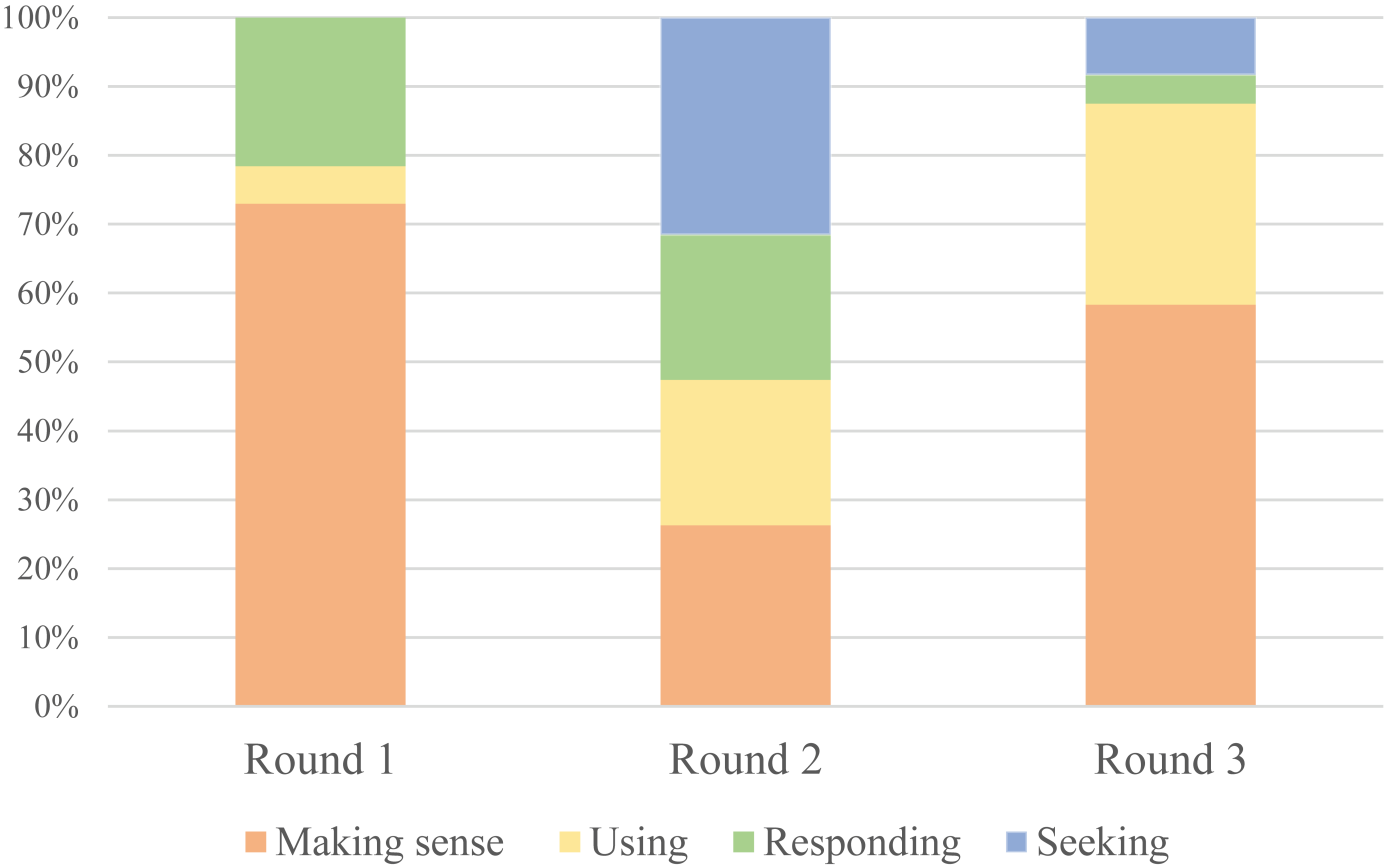
Learner evolving feedback process during SA. The learner’s engagement with feedback evolved in depth and proactivity across the self-assessment process. The composition of feedback activities shifted from a primary focus on understanding feedback to actively seeking it, indicating a developmental trajectory in feedback literacy. The sequential color gradient from orange to blue represents the increasing depth and proactivity of learner engagement with feedback.

Figure 3 illustrates the developmental trajectory of the student’s feedback process across the three SA rounds. In the first round, only three sub-processes were evident–making sense of feedback, using feedback, and responding to feedback–with making sense of feedback dominating (73%). This suggests that the student was still in the early stages of feedback literacy, focusing primarily on understanding rather than applying or internalizing the feedback.

In the second round, a notable shift occurred with the emergence of the “seeking feedback” sub-process–an important indicator of enhanced learner agency and interaction. According to Wang and Wang (2015), increased interaction correlates with greater learning effects, implying that the student’s learning potential had expanded in this round. All four feedback sub-processes were present in the third round, indicating a stable and mature feedback cycle. This suggests that the student had internalized the feedback process and was engaging in SA more autonomously and reflectively.

Collectively, this progression visualizes the gradual optimization of SA quality and the cultivation of feedback literacy through iterative SA activities. The student moved from a passive receiver of feedback to an active participant in the feedback loop, demonstrating increased metacognitive awareness and strategic self-regulation.

### 4.2 Visualization of cross-round interactions

Table 4 reveals a significant pattern: in Round 3, a considerable portion of self-feedback was inherited from earlier rounds, suggesting that the student had internalized prior feedback and was able to apply it independently in subsequent tasks. This aligns with the idea that external feedback serves as a scaffold that eventually supports the development of autonomous self-assessment skills.

**TABLE 4:** Source and target use of feedback in Round 3.

No.	Content from TAPs	Source	Target use
1	I should pay more attention to the tense.	Round 2 teacher feedback	Self-assessing
2	Do I need to add a definite article here? Yes, because it refers in particular to China.	Round 1 internal feedback and Round 2 teacher feedback	Translating
3	“The Chinese nation” should be a global concept, so the singular form is fine.	Round 2 teacher feedback	Translating
4	Plural forms should be used here just in case.	Round 1 internal feedback	Translating
5	The reference translation used present perfect tense here…	Round 1 teacher feedback	Self-assessing
6	In this translation task, there are many words that I do not know how to translate.	Round 1 internal feedback	Self-assessing
7	The translation of “全面建设小康社会” is fixed, and I should work on that later.	Round 2 internal feedback	Self-assessing
8	Using “it” to refer to “the road” is concise.	Round 1 teacher feedback	Self-assessing
9	If one concept is repeated many times in the original text, we can use “it” and “that” interchangeably to avoid unnecessary repetition.	Round 1 teacher feedback	Self-assessing
10	I did not perform well in expressions with Chinese characteristics, and I will accumulate similar expressions in the future.	Round 2 internal feedback and teacher feedback	Self-assessing

Moreover, the student demonstrated a clear ability to use feedback for both translation and self-assessment, indicating that the SA activities not only improved translation competence but also strengthened self-monitoring capabilities. Comparing the first drafts across Round 1 and Round 3, improvements were observed particularly in grammatical accuracy (singular/plural forms, tense usage), handling of long modifiers, and overall textual brevity.

Further evidence of learning was gathered through follow-up and final interviews (Segment 1 and Segment 2), where the student explicitly acknowledged her growth in SA and expressed a strong intention to continue using SA in future learning. These qualitative insights reinforce the conclusion that SA and feedback processes are mutually reinforcing, and that iterative SA activities can lead to both cognitive and metacognitive development in translation learning.

**Segment 1** (from follow-up interview)

*“This time around, I was able to do the self-assessment in a much more systematic way. I think having that framework from the first two rounds really made a difference. Looking back, it’s definitely more helpful for my translation learning than just error-checking like I used to do.”*

**Segment 2** (from final interview)

*“The biggest thing for me was developing a habit of self-assessment–you know, pausing to think over what I’ve learned and then using those insights in my next translation. I’d definitely like to do more of this in the future.”*

### 4.3 Learning effects of SA through exemplary learning chains

To provide concrete evidence of learning, two representative excerpts were extracted from the database and integrated into learning evidence chains (Excerpt 1 and Excerpt 2), which illustrate how SA facilitated the student’s translation development.

#### 4.3.1 Excerpt 1: self-feedback as a driver for learning

Evidence indicating internal feedback driving learning is shown in Excerpt 1.

**Excerpt 1** (Self-feedback for learning within round)

1. Original text of Task 3: *在上海的地铁里，上班族有的在打手机，有的在用笔记本电脑，有的在观赏车厢内显示器上播放的电影。全国各地，半导体工厂在昔日的农田拔地而起*.

2. First draft of Task 3: *In Shanghai’s* ***(1) railways****, some* ***(1) office people*** *are playing their phones, some are using their laptops and some are watching the film* ***(2) that is playing*** *on the screen. In China, the factories of* ***(1) conductors*** *are built on the places which was used to be farms.*

3. Reference translation of Task 3: ***Commuters*** *sitting on Shanghai’s* ***subway*** *talk on mobile phones, tap away on laptops or watch films* ***on*** *the train’s screen monitors. Around the country new* ***semiconductor*** *factories are shooting up on former farmland.*

4. Internal feedback:

*(1)* ***Insufficient vocabulary*** *leads to inaccurate rendition of some words;*

*(2) The translation is not* ***concise*** *enough.*

5. Revised draft of Task 3: *In Shanghai’s* ***(1)*** ***subways***, ***(1)*** ***commuters*** *are making phone calls, some are using their laptops, and some are watching the film* ***(2)*** ***played on*** *the screen. Around the country, factories of* ***(1)*** ***semi-conductors*** *are built on the places which were used to be farms.*

As shown in Excerpt 1, the student engaged in two internal feedback after reviewing the translation rubric and reference translation. The first internal feedback focused on vocabulary insufficiency, identified through comparison with the rubric. The second one highlighted the need for greater conciseness, derived from contrasting her draft with the reference translation.

The student then used these insights to revise her translation, selectively integrating expressions from the reference translation while retaining her own stylistic voice. This indicates a mature uptake of feedback, where the learner not only identifies gaps but also applies strategies to close them without fully imitating the model. This process also illustrates how multiple scaffolds (rubric, reference translation, and self-monitoring) interact synergistically to support high-quality self-assessment and learning outcomes.

#### 4.3.2 Excerpt 2: the complementary role of external feedback

Evidence that internal feedback alone is insufficient to drive certain aspects of learning is illustrated in Excerpt 2. The participant failed to recognize the error in translating “中国政府” as “China’s government” rather than “the Chinese government,” likely due to a belief that her rendition was acceptable. Only after receiving external feedback was this misconception corrected.

**Excerpt 2** (External feedback for learning across round)

1. Original text of Task 3: *现如今，中国政府正致力于扶持信息技术产业，使之成为一大经济支柱。*

2. First draft of Task 3: *Nowadays*, ***China’s government*** *is committed to support information and technology industry and intends to make the industry become the pillar of economic.*

3. Reference translation of Task 3: *Nowadays*, ***the Chinese government*** *is on a mission to make information technology a pillar of the economy.*

4. External feedback: *“China’s government” emphasizes a kind of subordinate relationship, which does not conform to the context of the original text; and the correct rendition of “中国政府” should be “the Chinese government.”*

5. Revised draft of Task 3: *Nowadays*, ***the Chinese government*** *is committed to supporting information and technology industry, and intends to make it a significant pillar of economy.*

6. First draft of Task 4:.*..Thanks to the advantage of Internet*, ***the Chinese government*** *is on the mission to…*

7. Revised draft of Task 4:.*..Therefore*, ***the Chinese government*** *will make great efforts to…*

Notably, the participant subsequently applied this knowledge in a later task (Task 4), accurately translating “中国政府” as “the Chinese government.” This indicates not only error correction, but also knowledge retention and transfer–key markers of deeper learning.

Collectively, these excerpts highlight how the interplay between internal feedback and external feedback–mediated through self-assessment (SA)–enhances translation learning. The learning chains illustrate how multi-source feedback, when processed via SA, contributes to greater linguistic accuracy, stylistic awareness, and conceptual understanding.

In summary, the findings of this study converge to show that self-assessment is not merely a summative exercise, but a dynamic and iterative process that cultivates feedback literacy, learner autonomy, and cognitive development. Through repeated SA cycles, learners gradually internalize feedback, refine translation strategies, and strengthen both their linguistic competence and metacognitive control. These insights hold important implications for designing translation instruction and assessment practices that support sustainable, learner-centered development.

## 5 Discussion

This study provides a foundational contribution to understanding Self-Assessment (SA) as a cognitive and metacognitive process in translation learning, particularly within China’s higher education context. By emphasizing the dynamic interaction between learner cognition and external scaffoldings, this research extends the theoretical understanding of SA and may offer insights for developing psychology-informed translation pedagogy. The findings advance the field in three key aspects: (1) demonstrating how structured scaffolding enhances translation performance through SA; (2) identifying the differential effects of feedback types on revision behaviors; and (3) empirically validating a conceptual model of SA-driven translation learning that integrates cognitive and metacognitive dimensions.

### 5.1 Self-assessment as a cognitive driver of translation development

The findings offer strong evidence that integrating SA into a structured translation learning environment serves as a powerful catalyst for linguistic and cognitive growth (To et al., 2022). Longitudinal analysis of the participant’s drafts revealed clear instances of linguistic transfer–for example, the successful adoption of the phrase “on the mission to” from a reference translation into later tasks (see Section “4.3.2 Excerpt 2: the complementary role of external feedback for details). These findings indicate that while exposure to high-quality reference translations prompts cognitive restructuring–a key feature of deeper learning–this process is initially stochastic. Through targeted practice, these nascent insights are consolidated into stable, internalized knowledge that constitutes translation learning.

Furthermore, while this study confirms the limited learning outcomes resulting from mere exposure to reference translations, it demonstrates that iterative self-assessment (SA) cycles–structured by consistent criteria and reflective prompts–enable learners to transcend this limitation. Through the internalization and transfer of insights across tasks, learners participate in a process consistent with the feedforward principle (Vardi, 2013). This finding highlights the importance of incorporating overlapping elements–such as terminology, syntactic patterns, and lexical diversity–into iterative SA activities, which collectively promote targeted and cumulative development while strengthening metacognitive awareness.

From a pedagogical standpoint, integrating self-assessment (SA) into translation instruction serves multiple purposes: it cultivates evaluative expertise, enhances metacognitive awareness, fosters learner autonomy, and deepens the understanding of translation quality standards–all of which are increasingly essential in the age of generative AI (Rivas Ginel and Moorkens, 2025). Accordingly, SA should be viewed not as a supplementary activity, but as a central component of a process-oriented assessment system in translation education.

### 5.2 Differential efficacy of feedback types in translation revisions

This study reveals a notable asymmetry in learners’ responses to different types of feedback. While feedback on surface-level issues–such as grammatical accuracy, tense consistency, and syntactic clarity–was generally incorporated into subsequent revisions, feedback targeting lexical diversity and systematic errors (e.g., article usage) was often acknowledged yet seldom implemented. This pattern aligns with the findings of Dressler et al. (2019) and Alharbi (2022), who noted that learners tend to prioritize revisions requiring lower cognitive effort over those demanding deeper conceptual engagement. These results thus enrich the Interaction Hypothesis by suggesting that while interaction may help learners recognize problems, it does not automatically lead to learning gains–especially for complex issues–and that targeted intervention is necessary in such cases.

Given these findings, an important question arises: How can translation instructors guide learners to effectively address global, meaning-level issues through scaffolded assistance? Two promising approaches emerge from the data. First, corpus-based and AI-supported scaffolding–for instance, using resources such as the Corpus of Contemporary American English (COCA)–can help learners internalize natural language patterns, thereby improving grammatical precision and lexical appropriateness. In addition, large language models (LLMs) such as ChatGPT and DeepSeek can effectively address multiple scaffolding-related challenges; for example, they can help evaluate the appropriateness of a learner’s word choice and provide abundant alternative expressions to support further translation development. Second, reflective learning journals–which task students with recording advanced vocabulary, collocations, and useful expressions–directly contribute to greater lexical variety and underpin the development of sustained translation competence. Beyond resolving immediate translation issues, this process simultaneously fosters learner autonomy and metacognitive awareness, thus aligning with overarching educational objectives that emphasize critical thinking and lifelong learning.

### 5.3 Unveiling the cognitive mechanism of SA in translation learning

**FIGURE 4:**
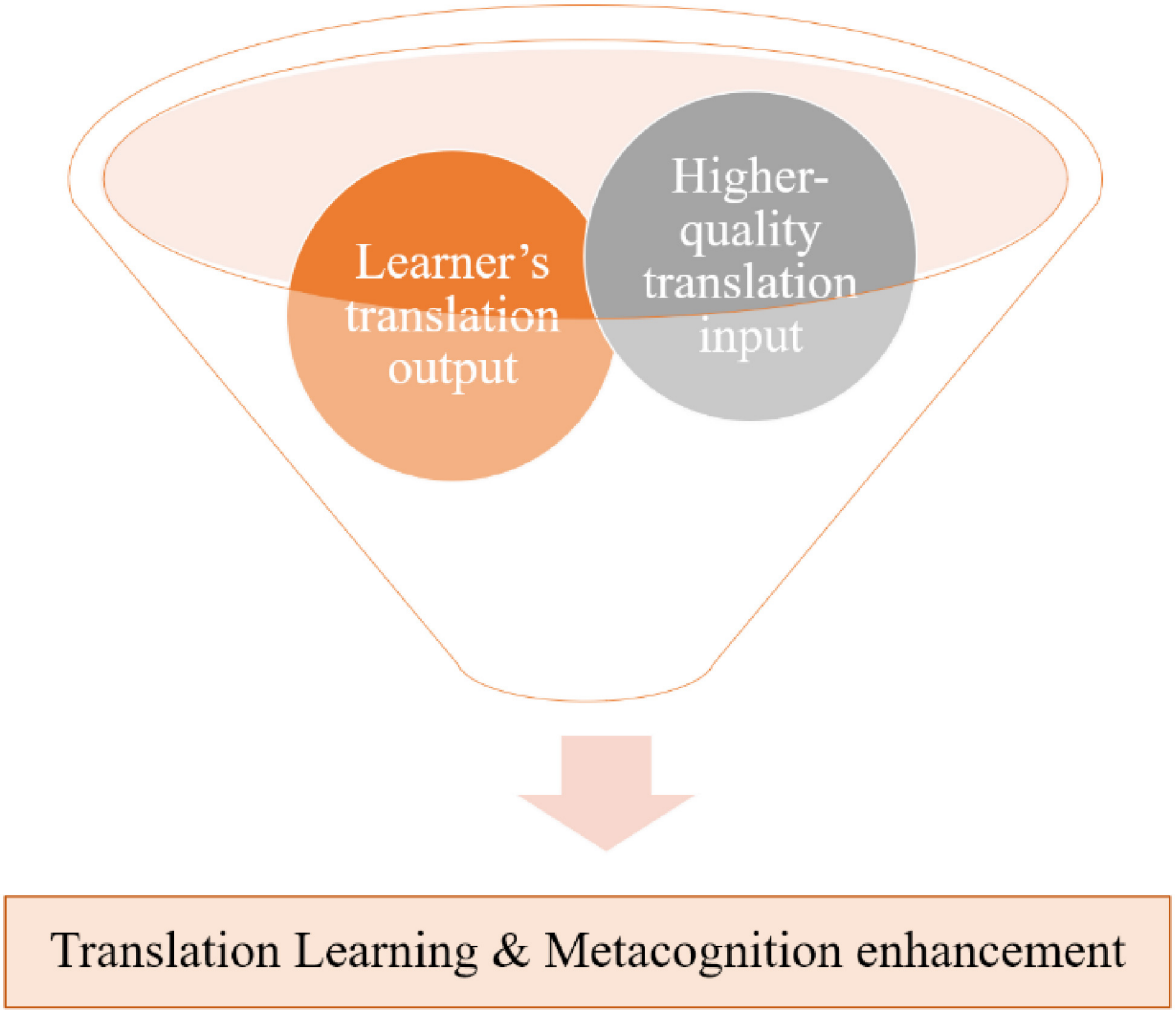
The “SA for translation learning” Funnel. This figure employs a funnel metaphor to illustrate how structured interaction between a learner’s translation output and high-quality input refines knowledge, thereby enhancing both translation competence and metacognitive awareness.

This study makes a key contribution by empirically validating a conceptual model of self-assessment (SA) in translation learning that centers on the comparative process between learners’ internal knowledge and external scaffoldings. As shown in Figure 4, the model demonstrates how a learner with strong translation competence and feedback literacy engage in cognitive dialogue–comparing their outputs with reference translations, rubrics, and external feedback–to refine their translation strategies.

These findings provide empirical support for Nicol’s (2021) claim that learners continuously employ comparative reasoning–a cognitive process shaped by prior knowledge, beliefs, and perceptions of feedback. Think-aloud data further confirm that the learner is capable of reflective and evaluative thinking, particularly when supported by structured scaffolding tools.

This study also corroborates De Kleijn’s (2021) feedback process model, evidencing the operation of its four reflective sub-processes. Notably, the feedback-seeking sub-process was not self-initiating; it depended on external scaffolding agents, such as instructors or potentially, prompt-engineered AI tools. This underscores the necessity of guided facilitation in the SA process, particularly in the initial phases of learner development.

The development of learners’ reflective depth and interaction quality across SA cycles supports Malecka et al.’s (2020) principle of practice. Importantly, it renders reflective quality a measurable element, supplying a validated indicator through which both learners and instructors can assess SA effectiveness and infer associated learning gains.

### 5.4 Refinement and validation of the conceptual model

**FIGURE 5:**
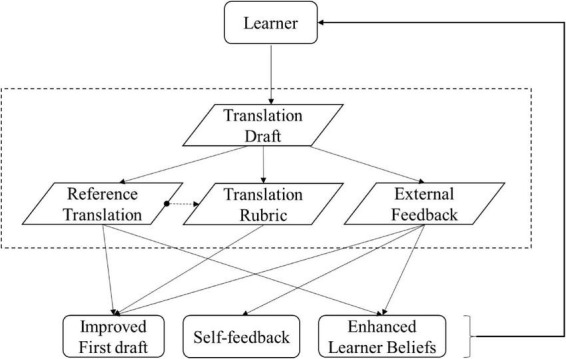
Detailed model of student self-assessment. The model reveals interactions among key learning elements. The dashed box encloses the self-assessment process, while rounded boxes indicate specific learning orientations achieved. Within this iterative cycle, the reference translation and rubric received simultaneous and focused attention from the learner, driving progressive improvement. All depicted interactions are supported by empirical evidence.

The initial conceptual model presented in Section “2.2.2 Synthesis and implications for current research” has been substantially refined and validated through the empirical findings of this study. As illustrated in Figure 5, the three core scaffoldings–reference translations, translation rubric, and teacher feedback–function as anchors that stimulate learner reflection and foster cognitive development.

Analysis revealed three key forms of learning evidence:

(1)    Improved translation drafts exhibiting higher linguistic and semantic quality;
(2)    Internalized feedback, which may not be immediately observable but supports long-term developmental gains;
(3)    Evolving translation beliefs, including the correction of prior misconceptions and the adoption of more professional norms and practices.

Furthermore, the study identified synergistic interactions among scaffoldings, particularly between reference translations and the rubric. This suggests that multi-modal scaffolding is more effective than the use of isolated one, aligning with Nicol and Selvaretnam’s (2021) assertion regarding the efficacy of rubric- and exemplar-based self-assessment.

Certain limitations in detectability were also noted, relating to individual learner differences and variations in the availability and quality of scaffolding materials. These factors may obscure more subtle or long-term learning outcomes, underscoring the need for future longitudinal research and investigations into individual variability in self-assessment processes.

In integrating cognitive science, metacognitive theory, and educational psychology, this study constructs a process-oriented model of self-assessment. This model is posited to contribute to both translation pedagogy and wider educational goals–including the promotion of mental well-being through structured, reflective practices that mitigate academic anxiety.

In summary, the findings advocate reconceptualizing self-assessment not as a static evaluation instrument, but as a dynamic, cognitively engaging process that promotes translation learning, professional growth, and lifelong reflective practice. Future research should investigate how self-assessment can be systematically integrated across diverse learner populations and educational settings, with particular emphasis on psychological support structures and technology-enhanced scaffolding.

## 6 Conclusion

In an era increasingly shaped by AI-driven translation tools, fostering students’ self-assessment (SA) and metacognitive abilities is essential. Overreliance on such technologies risks leading learners to conflate AI-generated outputs–which often surpass their own proficiency–with their personal translation competence, thereby undermining genuine academic and professional growth.

The case study advances the understanding of SA in translation by uncovering its cognitive-psychological mechanisms through the lens of the Interaction Hypothesis. The findings indicate that learning arises from metacognitive interactions triggered by discrepancies between high-quality external inputs (e.g., rubrics, reference translations, external feedback) and student-generated outputs. These interactions take the form of comparative processes–an internal feedback mechanism–through which learners conduct scaffolded self-assessment. The efficacy of learning is closely tied to the frequency and depth of such comparisons.

Theoretically, this study reframes SA as a dynamic belief–scaffolding interaction system, contributing to cognitive science and aligning with metacognitive and self-regulated learning (SRL) research. It extends the Interaction Hypothesis by conceptualizing comparison as a psychologically meaningful act that mediates between external supports and internal cognitive development. From an educational psychology perspective, the study proposes an optimized SA procedure aimed at enhancing feedback literacy and learner autonomy, while underscoring the possible role of task-relevant feedback in mitigating cognitive load and academic anxiety.

Practically, the findings support pedagogical designs that train learners to generate specific, comparative feedback–thus fostering both translational expertise and lifelong learning capabilities. This research also offers empirical validation for process-oriented assessment models in translation.

While this in-depth longitudinal case study, through its detailed analysis of a typical learner, has validated the proposed theoretical model and yielded rich pedagogical insights, future research could build upon its findings in several productive directions. Subsequent studies should (1) investigate genre-specific variations in the cognitive mechanisms of SA to test the generalizability of our model, and (2) incorporate multimodal data streams–such as eye-tracking and neurophysiological measures–to capture the fine-grained, in-situ dynamics of metacognitive processes during SA.

## Data Availability

The original contributions presented in this study are included in this article/Supplementary material, further inquiries can be directed to the corresponding authors.
