# Supplementary material for: A case study approach to the learning effects of self-assessment in translation learning: evidence and mechanism
Source: Front Psychol. 2026 Jan 8;16:1692773. doi: 10.3389/fpsyg.2025.1692773 (PMC12823955; doi:10.3389/fpsyg.2025.1692773)
Supplement: Supplementary file 1 [file Supplementary_file_1.docx]

**Appendices**

**Appendix A: Background questionnaire for the subject**

Name: _______ Matric No.: _____ Gender: _______ Age: ______

| 1. Duration of formal English education received: ______ years  2. College Entrance Exam (English score): ______ points  3. Duration of translation course study: ______ semester(s)  4. Final exam score of translation course in the last semester: ______ points  5. College English Test (CET) Band 4 score ______.  6. College English Test (CET) Band 6 score ______.  7. Test for English Majors (TEM) Band 4 score _____.  8. Test for English Majors (TEM) Band 8 score _____. |
| --- |

**Appendix B: Self-assessment rubric for translation**

| Score | Translation Features |
| --- | --- |
| 0 | -No understanding of the original text content at all.  -Plagiarism or direct copying of online translations or others’ translations  -Failure to complete the translation task, with 2/3 of the original text not translated. |
| 1 | -Very limited understanding of the original text.  -Major content errors.  -Low completion, missing important original text content.  -Very limited vocabulary, many spelling and punctuation errors, and inappropriate diction in many places.  -Many basic grammatical structure errors.  -Inability to properly use proper nouns or terminology.  -Sentence structures are rigidly copied from the original text. |
| 2 | -Able to understand the original text overall.  -Some content errors.  -Missing part of the original text content.  -Limited vocabulary, occasional basic vocabulary usage mistakes.  -Some basic grammatical errors, but mainly complex grammatical structure errors.  -Some proper nouns or terminology can be translated.  -Traces of literal translation, poor transfer between Chinese and English. |
| 3 | -Adequate understanding of the original text.  -Minor content is missing, but there may be distortion of the original text.  -The vocabulary is sufficient to complete the translation task.  -No basic grammatical errors, there may be problems in the use of complex sentences.  -A few improper translations of proper nouns or terminology.  -Basically, no traces of literal translation.  -Overall good translation expression effect. |
| 4 | -Understand the key and detailed information of the original text.  -No distortion of the original text.  -Although occasionally there is improper word choice, the vocabulary is rich and appropriate.  -Basically, no grammatical errors, and there may be one or two mistakes in complex grammatical structures.  -Translate proper nouns or terminology well.  -Demonstrate the awareness of discourse cohesion. |
| 5 | -Fully understand the key and detailed information of the original text.  -Clearly sort out the logical relationship of the original text.  -Effectively use a variety of advanced vocabulary to express the original text information.  -Grammatically accurate, almost no mistakes, rich and diverse sentence expressions.  -Can translate proper nouns or terminology very well.  -Accurately use a variety of cohesive devices, and the fluency is high. |

**Appendix C: Translation self-assessment questionnaire**

| **Part I: Self-Rating of the Translation Product**  Please score your translation script based on the self-assessment rubric for translation (Appendix B) and the reference translation: _____.  **Part II: Self-Reflection of the Translation Process**  (1) Sentence Completion Questions: Clearly state the strengths in your translation.  Note: Please complete the sentences by referring to this translation task, your own translation, the reference translation, and the scoring criteria. You can cite specific words or sentences from the original text, translation, or reference translation. Please see the examples (if the example fits your actual situation, it can also be filled in as an answer) and complete the following 5 sentences.  Examples:  -I think my strength lies in few grammatical errors and mistakes, which is due to post-translation checking and correction, and the reason prompting me to do so is that post-translation checks help improve the quality of the translation.  -I think my strength lies in appropriate vocabulary selection, which is due to I considered the context when choosing words, and the reason prompting me to do so is that I know whether it fits the context is one of the standards for measuring whether the word selection is appropriate.  1. I think my strength lies in __________, which is due to __________, and the reason prompting me to do so is __________.  2. I think my strength lies in __________, which is due to __________, and the reason prompting me to do so is __________.  3. I think my strength lies in __________, which is due to __________, and the reason prompting me to do so is __________.  4. I think my strength lies in __________, which is due to __________, and the reason prompting me to do so is __________.  5. I think my strength lies in __________, which is due to __________, and the reason prompting me to do so is __________.  (2) Open-Ended Short Answer Questions: Please use the original text, your own translation, the reference translation, and the rubric to carefully recall the translation self-assessment process and answer questions.  1. In the process of completing this translation task, what difficulties did you encounter that you could not resolve? What aspect do you think you should pay more attention to in subsequent translation learning? (Please write at least 3 points).  2. When evaluating your own translation, what is the part that you need to weigh repeatedly before making a decision (or what is the reason that you can’t grade your own translation)? (Please write at least 3 points). |
| --- |

**Appendix D: C-E translation tasks**

Task 3

在上海的地铁里，上班族有的在打手机，有的在用笔记本电脑，有的在观赏车厢内显示器上播放的电影。全国各地，半导体工厂在昔日的农田拔地而起。

这全都得益于中国拥有大量技术人才。依靠这些人才，中国的高科技设计和开发能力正日益提高。中国承担了大批全球制造业务，生产电视机、电脑、移动电话和其他电子产品。同时，中国正计划制造更复杂、精密的高科技产品。

现如今，中国政府正致力于扶持信息技术产业，使之成为一大经济支柱，重点发展半导体和软件工业，并对新公司采取提供税收优惠等激励措施。对外国投资者来说，中国的吸引力在于其规模庞大、扩展迅速的国内市场。另一个优势则是低工资，中国工程师的薪水仅是西方同行十分之一。

Task 4

互联网推进了中国经济社会发展。在经济领域，互联网加速向传统产业渗透，新型商务模式和服务经济加速兴起。互联网在促进经济结构调整、转变经济发展方式等面挥着越来重要的作用。

如今，互联网已成为推动中国经济发展的重要引擎。包括互联网在内的信息技术与产业，对中国经济高速增长做出了重要贡献。互联网发展与运用还催生了一批新兴产业，工业咨询、软件服务、外包服务等工业服务业蓬勃兴起。

正因如此，中国政府将大力推动电子商务类、教育类网站发展，支持发展网络广播、网络电视等新兴媒体，倡导提供形式多样、内容丰富的互联网信息服务，以满足人们多样化、多层次的信息消费需求。

Task 5

中华民族历经磨难，自强不息，从未放弃对美好梦想的向往和追求。实现中华民族伟大复兴的中国梦是近代以来中华民族的夙愿。

在新的历史时期，中国梦的本质是国家富强、民族振兴、人民幸福。我们的奋斗目标是，到2020年国内生产总值和城乡居民人均收入在2010年的基础上翻一番，全面建成小康社会。到本世纪中叶，建成富强、民主、文明、和谐的社会主义现代化国家，实现中华民族伟大复兴的中国梦。

实现中国梦，必须坚持特色社会主义道路。我们已经在这条道路上走了30多年。历史证明，这是一条符合中国国情、富民强国的正确道路，我们将坚定不移地沿着这条道路走下去。

**Appendix E: Reference translations**

Reference translation to Task 3

Commuters sitting on Shanghai’s subway talk on mobile phones, tap away on laptops or watch films on the train’s screen monitors. Around the country new semiconductor factories are shooting up on former farmland.

This is all thanks to China’s vast pool of technical brainpower, which is enhancing its high-tech design and development capabilities. It has taken over a large amount of global manufacturing, turning out television sets, computers, mobile phones and other electronic products. At the same time, it plans to produce more sophisticated high-tech products.

Nowadays, the Chinese government is on a mission to make information technology a pillar of the economy, targeting semiconductor and software industries and introducing incentives like tax breaks for new companies. China’s appeal for foreign investors comes from its huge and rapidly expanding domestic market. Another advantage is low wages. Salaries of Chinese engineers can be as low as one-tenth of those Western countries.

Reference translation to Task 4

The Internet is helping promote the economic and social development of China. In the economic sector, the Internet has spread its influence into traditional industry, which leads to the emergence of new business models and service economy. The Internet is playing an increasingly important role in promoting economic restructuring and transforming the pattern of economic development.

The Internet today has become an engine promoting the economic development of China. Information technology and industry including the Internet has made significant contributions to the rapid growth of the Chinese economy. The development and application of the Internet has given rise to the emergence of many new industries. Services for the development of industries such as industrial counseling, software service and outsourcing are mushrooming.

Therefore, the Chinese government will vigorously promote the development of websites featuring e-commerce and education, advocate the development of emerging media such as online radio and online television, and call for the provision of varied and of emerging media such as online radio and online television, and call for the provision of varied and rich Internet information services to satisfy the diversified, multi-leveled needs of information consumption.

Reference translation to Task 5

With great tenacity, the Chinese nation has emerged resilient from trials and tribulations. It has never given up its pursuit of great dreams. The great renewal of the Chinese nation has been a long-cherished dream of the Chinese nation since modern times.

In this new historical era, the Chinese dream that we are pursuing is about three things: economic prosperity, national renewal, and happiness of the Chinese people. We have two goals: First, to double the 2010 GDP and per capita income of both urban and rural residents and finish the building of a moderately prosperous society in all respects by 2020. Second, to build a modern socialist country that is strong, prosperous, democratic, culturally advanced and harmonious and fulfill the Chinese dream of achieving the great renewal of the Chinese nation by mid-21st century.

To realize the Chinese dream, we must stay on the path of socialism with Chinese characteristics. We have been on this path for over 30 years. History has proven it to be the right path that suits China’s national conditions, bringing about prosperity to China and its people. We will continue to advance along this path.

**Appendix F: Interview guide volunteers in the trial run of instruments**

C-E translation tasks

1. How difficult is this translation task for you?

2. Is this translation task appropriate for your current translation proficiency?

3. Is this translation task relevant to your short-term or long-term translation goals? Could you please explain?

4. How long did it take to complete this translation task?

5. Do you think you have benefited from completing the translation task? Why or why not?

6. Any additional comments?

Translation self-assessment questionnaire

1. Do you think this questionnaire helps assist you in performing self-reflection? Why or why not?

2. How long did it take to complete this questionnaire?

3. Do you have any suggestions on improving the overall design of the questionnaire to make it more user-friendly?

**Appendix G: Codebook for student feedback process**

| **No.** | **Nature of Code Development** | **Codes** | **Subcodes** | **Definitions** |
| --- | --- | --- | --- | --- |
| 1 | Theoretical | Making sense of feedback information | Orienting | What new content knowledge can I gain from this feedback information about this specific task? |
| 2 | Theoretical | Making sense of feedback information | Orienting | What new content knowledge can I gain from this feedback information that overarches this specific task? |
| 3 | Theoretical | Making sense of feedback information | Elaborating | How can I use the new content knowledge in this specific task? |
| 4 | Theoretical | Making sense of feedback information | Elaborating | How can I use the new content knowledge in other tasks? |
| 5 | Theoretical | Making sense of feedback information | Comparing | How does this feedback information compare to my self-assessment, to earlier feedback information and to other comparable works? |
| 6 | Theoretical | Making sense of feedback information | Comparing | What can I learn from these comparisons? |
| 7 | Theoretical | Making sense of feedback information | Identifying strengths | What does this feedback information tell me about the strengths of my work? |
| 8 | Theoretical | Making sense of feedback information | Identifying weaknesses | What does this feedback information tell me about points for improvement in my work? |
| 9 | Theoretical | Making sense of feedback information | Identifying unclarified | What feedback information remains unclear to me? |
| 10 | Theoretical | Making sense of feedback information | Using dialogue as an amplifier | Who can I discuss this feedback information with? |
| 11 | Theoretical | Using feedback information | Goal revision | Local: What goal(s) do I want to achieve within this specific task and how will I use this feedback information to reach this goal? |
| 12 | Theoretical | Using feedback information | Goal revision | Global: What goal(s) do I want to achieve in general and how will I use this  feedback information to reach this goal? |
| 13 | Theoretical | Using feedback information | Action planning | Time frame: What is the time frame for the use of my feedback information? |
| 14 | Theoretical | Using feedback information | Action planning | Activities: What do I have to do to use/apply the feedback information? |
| 15 | Theoretical | Using feedback information | Action planning | Standards: What standards do I want my work to meet? |
| 16 | Theoretical | Using feedback information | Action planning | Content: What content do I have to use? |
| 17 | Theoretical | Responding to feedback information | Synthesizing feedback information | What were the main points of the feedback information? |
| 18 | Theoretical | Responding to feedback information | Justifying changes made | What changes did I (not) make in response to the feedback information and why? |
| 19 | Theoretical | Responding to feedback information | Justifying changes made | Where in my document or performance can these changes be seen? |
| 20 | Theoretical | Responding to feedback information | Addressing emotional impact | What was the emotional impact of the feedback information? |
| 21 | Theoretical | Responding to feedback information | Addressing emotional impact | How did I handle this emotional impact? |
| 22 | Theoretical | Responding to feedback information | Use in follow-up assignments | How has earlier feedback information informed my current work? |
| 23 | Theoretical | Seeking feedback information: Follow-up feedback request | Synthesizing | What are the main points of the feedback information? |
| 24 | Theoretical | Seeking feedback information: Follow-up feedback request | Use of feedback | How and why did I use the feedback  information? |
| 25 | Theoretical | Seeking feedback information: Follow-up feedback request | Performance/  Product presentation | How is my feedback use reflected in my product/performance? |
| 26 | Theoretical | Seeking feedback information: Follow-up feedback request | Emotional impact | What was the emotional impact of (working with) the feedback information? |
| 27 | Theoretical | Seeking feedback information: Follow-up feedback request | Requesting feedback information | ‘Could you give me feedback on the extent to which my product or performance has improved as a result of my feedback use?’ |
| 28 | Theoretical | Seeking feedback information: Problem-based feedback request | **P**roblem description | What problem do I encounter? |
| 29 | Theoretical | Seeking feedback information: Problem-based feedback request | **O**ption overview | What options do I see for solving this problem? |
| 30 | Theoretical | Seeking feedback information: Problem-based feedback request | **W**eighing options | How do I weigh these options in terms of pros and cons? |
| 31 | Theoretical | Seeking feedback information: Problem-based feedback request | **E**xpressing preferred option | Given these pros and cons, what option(s) would I chose myself? |
| 32 | Theoretical | Seeking feedback information: Problem-based feedback request | Requesting feedback information | ‘Could you give me feedback on this (i.e. elements P, O, W, and E)?’ |
| 33 | Theoretical | Seeking feedback information: Learning objective-based feedback request | Current performance | My current performance, does (not) reflect the best I could do without additional assistance, because… |
| 34 | Theoretical | Seeking feedback information: Learning objective-based feedback request | Learning Objective | I would like my work to be evaluated on the following learning objective(s)… |
| 35 | Theoretical | Seeking feedback information: Learning objective-based feedback request | Self-Evaluation | This is what I think I did well and what I would need to further improve |
| 36 | Theoretical | Seeking feedback information: Learning objective-based feedback request | Requesting feedback information | ‘Could you give me feedback on this (i.e. elements C, LO , and SE)?’ |

## Appendix H: Trial results of research instruments

Table 1. Trial results of two translation tasks

| No. | Volunteers | Translation tasks | Feedback |
| --- | --- | --- | --- |
| 1 | Third-year undergraduate | Task 2 | *30 minutes are sufficient to complete the task. It is neither difficult nor easy for me, and I can learn something.* |
| 2 | Four-year undergraduate | Task 1 | *I finished the task in 20 minutes. Although the translation task was easy, I still learned a lot.* |
| 3 | Second-year undergraduate | Task 2 | *I find no difficulty finishing the task in 30 minutes, and the task is a little easier than some tasks I encountered. But I still make many mistakes when doing it, so I think the task is proper for me overall.* |
| 4 | First-year undergraduate | Task 1 | *I find the topic of the task familiar and intriguing, and I can complete it in less than twenty minutes.* |

Table 2. Trial results of the Translation Self-Assessment Questionnaire

| No. | Volunteers | Feedback |
| --- | --- | --- |
| 1 | Third-year undergraduate | *The overall design helps deepening my reflection; providing reflection examples for each open question will be better.* |
| 2 | Four-year undergraduate | *The questionnaire improves my self-assessment awareness. I prefer a completion-based reflection to open questions.* |
| 3 | Second-year undergraduate | *The reflection examples provided are abstract in expression, which exceeds my cognitive level, thus impeding students from writing their ideas freely.* |
| 4 | First-year undergraduate | *The hints in the questionnaire should be changed from statements to questions for clarity. In contrast to the ease of reflecting on my weaknesses, expressing my strengths is more complicated. With the combined use of reference translation and a self-assessment rubric, I am confident in my self-rating.* |
| 5 | First-year undergraduate | *“Revising future learning goals” should be put after “weaknesses”. Although the examples are helpful, filling out a blank table is still not easy, so I wonder if more hints could be given to make the blank table a cloze-like one.* |

**Appendix I: Three-round first draft and revised draft**

| **Round 1** | |
| --- | --- |
| First draft | Revised draft |
| In Shanghai’s railways, some office people are playing their phones, some are using their laptops and some are watching the film that is playing on the screen. In China, the factories of conductors are built on the places which was used to be farms.  It is because there are lots of technolog talents in China. With these talents, China’s design of advanced technolog and the ability of investing is improving increasingly. China shoulders a large number of global manufacturing orders, producing televisions, computers, mobile phones and other electronic devices. In the meanwhile, China is planning to making advanced productions that are more complex and concise.  Nowadays, China’s government is committed to support information and technology industry and intends to make the industry become the pillar of economic. And China focuses on developing conductor and software industry as well as taking encouraging measures for new companies such as offering tax benefits. For foreign investors, there are two advantages of China that interest them to invest. One is that China’s domestic market is large and expanding rapidly, the other is the low wage. In China, the salary of the engerienner is only the one tenth of that in Western. | In Shanghai’s subways, some office people are making phone calls, some are using their laptops, and some are watching the film played on the screen. Around the country, factories of semi-conductors are built on the places which were used to be farms.  It is because there are lots of technological talents in China. With these talents, China’s design of advanced technology as well as the development capacity are improving increasingly. It shoulders a large number of global manufacturing orders, producing televisions, computers, mobile phones and other electronic products. At the same time, it is planning to make advanced products that are more sophisticated.  Nowadays, the Chinese government is committed to supporting information and technology industry, and intends to make it a significant pillar of economy. It also focuses on developing semi-conductor and software industry, and carries out incentives such as providing tax breaks for new companies as well. For foreign investors, China’s appeal lies in its domestic market with large scale and high speed of expansion. The low salary is another advantage. The salaries of Chinese engineers are only the one- tenth of those in Western. |

| **Round 2** | |
| --- | --- |
| First draft | Revised draft |
| Internet has promoted the development of China’s economic society. In the field of economy, traditional industries accelerate to combine with Internet, and new business models as well as service economy are rising up with high speed. Internet plays a more and more significant role in promoting the regulation of economic structure and the transformation of economic development method.  Nowadays, Internet has become important engines for pushing on China’s economic development. Information technologies and industries including Internet make great contributions to the high-speed development of China’s economy. The development and application of Internet also give rise to a new round of new industries, with service industry such as industrial consultation, software service and asking the third party to takeover services developing rapidly.  Thanks to the advantage of Internet, the Chinese government will take its efforts to promote the development of electronic businesses and educational websites, support the development of new media such as online brocasters and online televisions, encourage to provide Internet information services with multiple forms and abundant contents to meet the people’s needs for diverse and multi-side information consumption. | The Internet has promoted the development of China’s economy and society. In the economic field, the Internet is accelerating to influence traditional industries, with new business models and service economy emerging rapidly. The Internet has played an increasingly significant role in promoting economic restructuring and transforming the economic development pattern.  Nowadays, the Internet has become an important engine to promote the economic development of China. Information technology and industry including the Internet has made great contributions to the rapid growth of China’s economy. With the development and application of the Internet, services for the development of industries such as industrial consulting, software service and outsourcing rise up in a great number.  Therefore, the Chinese government is on a mission to promote the development of websites for electronic commerce and education, support the development of new media such as online broadcasting and televisions, and encourage to provide Internet information services with multiple forms and abundant contents to meet people’s various and multi-leveled needs of information consumption. |

| **Round 3** | |
| --- | --- |
| First draft |  |
| Though the Chinese ethnic has been through a hard time, they never give up their pursuit of wonderful dreams. Chinese dream to realize the great recovery of the Chinese ethnic has become the dream of the Chinese ethnic since modern China.  In the new period of history, the nature of Chinese dream is the prosperity of the nation, the development of the Chinese ethnic and the happiness of people. Our goal is that the GDP and people’s average income in 2020 is 2 times of those in 2010, and to fully develop a prosperous society. In addition, in 2050s, we plan to build a modern socialism country that is prosperous, democrative, civilized and harmonious to realize the Chinese dream.  If we want to realize the Chinese dream, we must be committed to the road of socialism with Chinese characteristics. We have adhered to the road for 30 years. History proves that the road of socialism with Chinese characteristics is suitable for Chinese situations, and is a correct road to make people wealthy and China powerful. Therefore, we will insist on this road. |  |

**Appendix J: Three-round self-feedback summary**

| **Round 1** |
| --- |
| 1. Many words are challenging for me, such as “semiconductor factories,” “carriages,” “commuters,” “development capabilities,” etc.  2. I lack flexibility in handling certain sentence structures. For example, the reference translation’s first sentence is concise, but I broke it down into three short sentences, indicating there is significant room for improvement. I should study more translation examples and accumulate learning experiences.  3. Translate the core part of the sentence first: The reference translation uses “commuters” as the subject, with “sitting” as a complement, which is similar to what I learned in textbooks; namely, first look at the sentence, then analyze what is background, what is supplementary information, and what is the core.  4. My issue is that my expressions are neither idiomatic nor concise.  5. Pay attention to detaching from the form of the original text, that is, to de-shell it, to distinguish between important and secondary information, and to focus on the key points.  6. Avoid overusing relative clauses, as they can make the text verbose when used excessively.  7. Conciseness in word choice: If one word can express the meaning, don’t try to use two.  8. Pay attention to singular and plural forms.  9. I also don’t know how to express fractions.  10. The goal of translation is to achieve idiomatic and concise expression. |
| **Round 2** |
| 1. It is not difficult for me to comprehend the original text, as I have encountered similar articles and have a certain understanding of the Internet. It is necessary to keep up with current events and learn the development of various industries in China.  2. My translation is relatively fluent and coherent. I pay great attention to logic and coherence in my writing, because the logic of writing is crucial to the comprehensibility of English texts.  3. My current vocabulary can roughly cope with this translation task. I have been memorizing words regularly. A large amount of vocabulary is the foundation of good translation.  4. There are few grammatical errors. I am particularly careful not to make grammatical mistakes, as they can greatly reduce the quality of the translation and are basic errors.  5. There is basically no missing information in my translation. I have learned that translation should mainly use direct translation and must not delete information from the original text arbitrarily, as that is a dereliction of duty for a translator.  6. I am not sure how to translate some words, so I should read more and accumulate more expressions with Chinese characteristics in my daily life.  7. There is a deviation in understanding, and I cannot determine whether my understanding is correct or not. For understanding issues, I still need to practice more.  8. I hesitate over the use of singular and plural forms, such as “traditional industries,” “information technology industry.” Sometimes I use the singular form where I should use the plural and vice versa. This encourages me to pay more attention to this small detail in future translation studies.  9. It is challenging to increase the richness of vocabulary. For example, the original text mentions “推进” multiple times, and I have been trying to express it with a variety of words, but what comes to my mind is limited.  10. When evaluating the strengths of my translation, I find it difficult to think of strengths because I focus more on shortcomings, and I am not sure whether they are strengths.  11. I am not sure whether my translation level is level 2 or 3 because my translation meets some of the characteristics of both levels.  12. For expressions with Chinese characteristics, I am not sure whether it is necessary to follow a fixed translation or if it is also acceptable to express it in my own words? |
| **Round 3** |
| 1. I accidentally omit the preposition “over”.  2. Many expressions were not translated or were translated incorrectly, such as “国情,” “翻一番,” “人 均,” and “城乡居民.”  3. Adjusting and clarifying sentence structure or logic.  4. Position of temporal adverbials: The reference translation places the temporal adverbial at the end, but I think placing it earlier is also a good choice.  5. Pay attention to the correct use of tenses; the present perfect tense is more appropriate for this text.  6. Using demonstrative pronouns to refer to repeatedly mentioned nouns can enhance the conciseness of the translation.  7. Sentence structures: Sentences can be flexibly handled; information that belongs to the same category can be grouped together and summarized with a leading sentence for simplification.  8. Regularly accumulate commonly used expressions with Chinese characteristics.  9. Use punctuation effectively: For parallel elements, a colon can be used.  10. If the attributive clause is too long, it is generally placed at the end (post-modification).  11. Choose words carefully: use the best one.  12. My translation is a bit lengthy.  13. My translation is relatively smooth.  14. There are not many grammatical errors.  15. In the future, I should pay attention to being as faithful to the original text as possible; the meaning of the original text should be fully conveyed. |

**Appendix K: Two-round teacher feedback summary**

| **Round 1** |
| --- |
| 1. The richness of vocabulary needs to be improved.  2. The understanding of the original text needs to be deepened (e.g., “打手机”).  3. Use pronouns flexibly to achieve coherence (e.g., using “it” to replace “China”).  4. The accuracy of word choice needs to be improved (e.g., “electronic products”).  5. Misuse of verb forms following some fixed phrases (e.g., “be committed to doing”).  6. The distinction between “economy” and “economic.”  7. Some elements can be omitted to enhance the conciseness of the translation (e.g., “to invest”).  8. One or two grammatical errors (e.g., “that in Western”).  9. The overall translation is good with no missing information. |
| **Round 2** |
| 1. From a diachronic perspective, this round entails more detailed feedback, which is great.  2. The understanding of “工业服务业” needs to be improved. That Chinese phrase refers to industries that serve the industry.  3. You can generally understand the original text, but there is a lack of understanding of some professional vocabulary. If you encounter unfamiliar vocabulary later, you should try to associate it with existing knowledge to interpret and solve the problem of appropriate word choice, such as between “method” and “pattern.”  4. The expression is smooth, but there is an overuse of “of” structure. Sometimes, an adjective plus a noun can clearly convey the meaning, and in this case, there is no need to use an inverted structure because using “of” increases the length of the translation and also makes the sentence structure unnecessarily complex.  5. The use of tense, number, and articles is slightly chaotic. Translation learning requires developing sensitivity to tense and number markers, such as “了” and “等.”  6. Yes, “等” also needs to be translated, and it can also be translated as “such as,” because “等” itself carries a plural marker.  7. Abstract concepts are actually conceptualized nouns, such as “Internet,” “new business models,” “service economy.”  8. It is acceptable to add an “s” to “包括互联网在内的信息技术.”  9. There are some missing minor details, such as “vigorously” in “vigorously promote e-commerce.”  10. The use of verb phrases needs to be improved, for example, “use efforts to” is incorrect.  11. You need to check the structure of long sentences because they are the most prone to errors.  12. In a parallel structure, the second verb phrase does not need to add “to” again.  13. The best way to improve vocabulary richness is to systematically accumulate synonymous expressions.  14. Expand the vocabulary memory unit, such as remembering word strings rather than individual words or phrases.  15. For your question, my response is that the best option is that you know its fixed translation, but if you really do not know the fixed translation, translating the meaning is also acceptable. |

**Appendix L: Dos’ and Don’ts list generated by the subject**

| **Round 1** |
| --- |
| 1. Increase vocabulary by actively accumulating words and expressions, such as “subway,” “carriage,” “semiconductor,” “development capability,” “sophisticated,” “tax incentives,” etc.  2. For unfamiliar words, use association to aid understanding.  3. Improve the coherence of the translation.  4. Avoid repetitive vocabulary.  5. Pay special attention to the use of singular and plural forms. If not referring to a specific individual, generally use plural forms, and when using singular, pay attention to the use of articles.  6. Do not make spelling mistakes with words; I misspelled “engineer” and “technology.”  7. Never omit information; even the smallest details should be translated.  8. Flexibly use “as,” “with,” and “-ing” to connect sentences and enrich sentence structure. |
| **Round 2** |
| 1. Pay attention to tenses and be mindful of the tense markers in the original text. However, I have a question: Is it necessary to unify the tenses? The translation uses the perfect tense while I used the present continuous tense. Are both acceptable, or is one better than the other?  2. Memorize phrases as a whole. If you have difficulty understanding when translating, use association to draw upon your learned knowledge.  3. For singular and plural forms, there’s no need to be overly concerned. General/abstract: use singular forms; specific/multiple: use plural forms. Moreover, you can use the semantics of the original text to help choose between singular and plural.  4. When multiple expressions are available, choose the most concise one, such as “at a high speed” and “rapidly.”  5. It’s important to accumulate and memorize expressions with Chinese characteristics.  6. Skillful use of non-finite verbs, “with” structures, and “as” can enrich sentence patterns, but also be careful not to omit or mistranslate.  7. I wasn’t sure how to translate “蓬勃兴起” (thriving), so I translated it based on my understanding as “emerged in large numbers.” I’m not sure if that’s acceptable. The reference translation’s use of “mushrooming” seems ingenious.  8. “正因如此” (That’s why) initially had me pondering how to connect it with the preceding text, but the translation resolved it with a single “therefore”. It’s important to learn to be flexible in dealing with such situations.  9. For long sentences, pay attention to grammar and coherence.  10. Regarding modifiers like “形式多样,内容丰富” (diverse forms, rich content) and “多样化,多层次的” (diverse and multi-level), I intended to process them as pre-attributives to make the translation more concise. However, due to not thinking of a single adjective with multiple meanings, I ended up processing them as postpositional attributives. I should remember this for future use. |

**Appendix M: TAP(s) training materials**

1. Warming-up

(1) Please name ten types of food you might find in a supermarket.

Okay. As you come up with the answer to this question, do you have any other thoughts? I want you to say out loud whenever these thoughts occur to you. Do not explain your thoughts to others. Just say what comes to your mind—even if it’s not always grammatically correct. Listen to the next question and think of the answer as quickly as possible! Are you ready?

(2) What is the sixth letter after the letter “C”?

Okay. Upon hearing this question, you might not immediately think of the letter “I.” You might need to go through several steps to find the answer. If you summarized your thoughts rather than reporting the actual sequence of thoughts out loud in response to this question, you might say that you found the letter “I” by counting through the alphabet. But that’s not what I’m asking you to do, because it misses the sequence of thoughts. When people genuinely solve this problem through think-aloud protocols, they typically verbalize a series of individual letters before arriving at “I,” such as C, then D, E, F, G, and H. Similarly, that’s what I would like you to do, because we’re interested in the thoughts you have as you answer the question; we’re looking for the most accurate, detailed report of your thoughts as possible, not a summary of those thoughts.

2. Translation tasks and reference translation for training TAP

- 河南是中国重要的经济大省，2017国内生产总值稳居中国第5位。2017年河南生产总值44,988亿元，比上年增长7.8%，人均生产总值47130元，增长7.4%。

Henan is China’s major provincial economy. Its GDP of 2017, fifth in China, was 4.4988 trillion yuan, up by 7.8% over the previous year. In terms of its GDP per capita, the two figures were 47,130 yuan and 7.4% respectively.

- 健康是促进人的全面发展的必然要求。提高人民健康水平，实现病有所医的理想，是人类社会的共同追求。在中国这个有着１３亿多人口的发展中大国，医疗卫生关系亿万人民健康，是一个重大民生问题。

Good health is a prerequisite for promoting all-round development of the person. And it is a common pursuit of human societies to improve people’s health and ensure their right to medical care. For China, a large developing country, medical and healthcare is of vital importance to its population of over 1.3 billion, and is a major issue concerning its people’s well-being.

- 能源安全同世界经济的稳定发展和各国人民的福祉息息相关。在当前国际金融危机背景下，维护全球能源安全对有效应对国际金融危机冲击、推动世界经济全面复苏和长远发展具有重要意义。

Energy security has a close bearing on the stability and growth of the world economy and the well-being of people in all countries. Against the backdrop of the global financial crisis, ensuring energy security is vital to tackling the impact of the crisis and promoting the full recovery and long-term development of the world economy.
